# High-dose omega-3 polyunsaturated fatty acid supplementation might be more superior than low-dose for major depressive disorder in early therapy period: a network meta-analysis

**DOI:** 10.1186/s12888-020-02656-3

**Published:** 2020-05-20

**Authors:** Xu-dong Luo, Jin-shan Feng, Zheng Yang, Qiao-ting Huang, Ju-da Lin, Bo Yang, Kuan-pin Su, Ji-yang Pan

**Affiliations:** 1 Department of Psychiatry, The First Affiliated Hospital, Jinan University, Guangzhou, 510630 China; 2 Department of Psychiatry, Affiliated Hospital of Guangdong Medical University, Zhanjiang, 524001 China; 3 Marine Medicine Research Institute, Guangdong Medical University, Zhanjiang, 524023 China; 4 Institute of Lipids Medicine and School of Public Health, Wenzhou Medical University, Wenzhou, 325035 China; 5 Department of Psychiatry, China Medical University Hospital, Taichung, 400 Taiwan

**Keywords:** Omega-3, Polyunsaturated fatty acid, Supplementation, Major depressive disorder, Network meta-analysis

## Abstract

**Background:**

The application of n-3 Polyunsaturated Fatty Acids (n-3 PUFAs) supplementation for major depressive disorder (MDD) has been widely discussed in recent years, but its efficacy and application are still controversial. This network meta-analysis was conducted to compare the efficacy of different dosages of n-3 PUFAs on MDD patients in the early period of treatment.

**Methods:**

Randomized controlled trials (RCTs) exploring the efficacy of n-3 PUFA supplementation for patients with MDD were retrieved from the databases of Pubmed, Embase and the Cochrane Library. RCTs comparing the efficacy of n-3 PUFA for adult (≥18 years) MDD patients without comorbidity were eligible for our study. The score of depressive symptoms in early therapy period of the treatment (≤9 weeks) was extracted. Standardized mean deviations (SMDs) of all the sores from the eligible RCTs were synthesized in a pairwise meta-analysis in frequentist framework and a random-effects network meta-analysis in Bayesian framework for the overall and subgroups (high- and low-dose) efficacy of n-3 PUFAs.

**Results:**

A total of 910 MDD patients in 10 trials with 3 adjuvant therapy strategies (high-dose n-3 PUFAs, low-dose n-3 PUFAs and placebo) were included. Results of pairwise meta-analysis showed that n-3 PUFAs were superior to placebo (SMD: 1.243 ± 0.596; 95% CI: 0.060 ~ 2.414). Results of the network meta-analysis showed that both the high (SMD: 0.908 ± 0.331; 95% CI: 0.262 ~ 1.581) and the low-dose (SMD: 0.601 ± 0.286; 95% CI: 0.034 ~ 1.18) n-3 PUFAs were superior to placebo, and the efficacy of high-dose n-3 PUFAs is superior to that of low-dose.

**Conclusions:**

High-dose n-3 PUFAs supplementation might be more superior than low-dose in the early therapy period for MDD. More head-to-head clinical trials need to be carried out to provide more direct comparison and enhance the evidence of the efficacy of n-3PUFAs for MDD.

## Key findings

High-dose n-3 PUFAs supplementation is more superior than low-dose for MDD patients.

## Background

Omega-3 polyunsaturated fatty acids (n-3 PUFAs) are essential fatty acids for the human body as they are unable to be synthesized in vivo but need to be obtained from the diet. Long chain n-3 PUFAs, mainly including eicosapentaenoic acid (EPA) and docosahexaenoic acid (DHA), play an important role in transport of multiple ions across the cell membrane where there is a need for stable permeability and fluidity. These characteristics can be maintained in an optimal state by the n-3 PUFAs. In recent years, it has been found that patients among various types of depression [1–4] with a lower level of DHA and (or) EPA, so it is considered to be used as an adjuvant treatment of the major depressive disorder (MDD) theoretically. Clinical trials revealed that n-3 PUFAs improve depressive symptoms. However, the previous meta-analysis found the efficacy of n-3 PUFAs for MDD patients was controversial [5, 6]. Up to now, the standard dosage for n-3 PUFA in depression adjuvant therapy is not yet established. Does DHA and (or) EPA levels in MDD patients decrease more severely? Whether MDD patients need higher doses of n-3 PUFAs remains to be further explored. Su et al. revealed that a high dose of n-3 PUFAs (4400 mg EPA + 2200 mg DHA per day) had a significantly decreased score on the 21-item Hamilton Depression Rating Scale (HDRS) [7]. However, the effects of a high dose of DHA on depression are still unclear [8–10], because most of the evidence supports the theory of EPA superiority [11, 12]. Before effective doses of n-3 PUFA were recommended based on the results of head-to-head trials for MDD patients, comparative effectiveness research is necessary to identify the efficacy of different doses of n-3 PUFA supplementation. So a more rigorous analytical approach needs to be applied. In this study, we used both the pairwise and network meta-analysis to compare the efficacy of high-dose and low-dose n-3 PUFAs for MDD patients and to find out a relative superior strategy for clinical decision-making. In summary, we have noticed there were still several issues to be solved: (1) The effective dose of n-3 PUFAs for MDD is not yet clear, EPA dose in different clinical trials varies greatly (from 45 to 4400 mg/day), whether the efficacy of high-dose n-3 PUFAs is more superior than that of low-dose are not yet identified, and the effect of dose variation on therapeutic efficacy needs to be explored. (2) Whether the efficacy of n-3 PUFAs for MDD was affected by the quantity of DHA needs to be explored. (3) Heterogeneity of included trials needs to be further explored, as the eligible participants, antidepressants usage, dosage and duration of n-3 PUFAs administration, and research duration may be correlated with the estimated results.

## Methods

### Eligibility criteria

Randomized controlled trials (RCTs) comparing the efficacy of n-3 PUFAs supplementation with/without placebo for MDD patients were eligible, inclusion and exclusion criteria for RCTs were as follows.

#### Inclusion criteria

(1) Studies designed as RCT. (2) Adult (≥18 years) eligible patients of MDD were all diagnosed according to the Diagnostic and Statistical Manual IV.

#### Exclusion criteria

(1) trials specifically studied perinatal or perimenopausal MDD, (2) trials included MDD patients with comorbidity, (3) outcome data were unable to be obtained from the literature or by contacting the authors.

### Information sources and search strategy

Literature was searched in the databases of MEDLINE, EMBASE and Cochrane Library. The publication year and language were not restricted. The search strategies were provided in Supplementary method.

### Data collection

Data of primary outcomes in eligible literature were extracted by two authors independently. Meanwhile, other information such as the first author, publication year, population characteristics, treatment duration, DHA dose, baseline severity were also collected. If patients enrolled in some trials were not all MDD (such as mild-to-moderate or moderate-to-severe depression), the documented information was judged particularly according to the diagnosis criteria of depression and baseline severity of depression. After discussed by all the authors, that literature was decided whether to be included. To reduce heterogeneity and improve the accuracy of our analysis, we only collect the data of primary outcome within 9 weeks as most of the research duration of included literature contained outcome-data in week 8. To make full use of the published data, if there were two arms of high- or low-dose n-3 PUFAs existed in one three-arm studies, we combined the sample size, mean and standard deviation respectively into one arm according to the method provided in the Cochrane handbook [13].

### Risk of bias assessment

Risk of bias for individual and across the included RCTs were assessed by the Cochrane Risk of Bias assessment tool in Revman software (version 5.3) from the aspect of selection, performance, detection, attrition, reporting and other bias.

### Summary measure and synthesis of results

A pairwise meta-analysis by the subgroup of different dose n-3 PUFAs was conducted using the STATA software (version 15). Based on the guidelines of International Society for Nutritional Psychiatry Research Practice (ISNPRP), a dose of EPA net content of 1000–2000 mg/day is recommended for major depressive disorder [12]. EPA or (and) DHA greater than or equal to 2000 mg/day was included in the high dose n-3 PUFAs subgroup, otherwise included in the low-dose n-3 PUFAs subgroup in this analysis. Both the high- and low-dose n-3 PUFAs subgroup was compared with placebo subgroup. A Bayesian network meta-analysis was conducted by using the Markov chain Monte Carlo method in the R software (version 3.6.1) which has been introduced previously [14]. The surface under the cumulative ranking curve (SUCRA) was used to rank the probability of the best one between all the interventions by comparing with each other [15]. Results of SUCRA range from 0 to 1, the greater the value, the more effective the strategy is [16]. Given the expected heterogeneity, we used a random-effects model for all methods used in results synthesis, and the results were reported as mean and 95% confidence interval (CI).

### Additional analyses

#### Heterogeneity

The judgment of heterogeneity refers to the results of the *I*^*2*^ statistic and *P* statistic of the *Q* test. Statistical heterogeneity exists when *I*^*2*^ statistic is more than 50%, or when *P* statistic less than 0.1 and *I*^*2*^ more than 25% at the same time. Sensitivity analyses were carried out for each subgroup using the STATA software (version 15). Network meta-regression were performed using the R software (version 3.6.1) with covariates of the publication year, DHA dose, time for treatment, baseline severity in the Bayesian framework.

#### Publication bias

Publication bias was investigated using Egger’s regression and Trim-and-Fill method for each subgroup in the STATA software (version 15).

## Results

### Study selection

A total of 214 pieces of literature were identified with the search strategy, 76 pieces of literature were excluded according to the results of duplication-checking, 67 unrelated pieces of literature were excluded through the analysis of title and abstract, 61 pieces of literature were excluded according to exclusion criteria. a total of 10 trials [7, 9, 17–24] with 3 strategies (high-dose n-3 PUFAs, low-dose n-3 PUFAs and placebo) containing 910 patients were included in this analysis (Supplementary figure 1).

### Study characteristics

Nine of the 10 studies were designed as placebo-controlled. Three of 10 studies were three-arm research (EPA vs DHA vs placebo [22, 23], EPA vs DHA vs EPA + DHA [24]) Nine of the 10 articles included were MDD patients, only one study [22] with mild-to-moderate depression patients were included in our analysis, in which patients met the criteria of MDD, and the baseline severity of MDD was comparable to other included studies. But another study [25] with moderate-to-severe depression patients were excluded as the exact number of MDD patients were unable to be acquired. In addition, one study was excluded because its outcomes data were not reported in the form of the mean and standard deviation [24]. Five included studies [7, 9, 17, 18, 24] were with high-dose PUFAs treatment and the rest were low-dose n-3 PUFAs. The characteristics of included RCTs were shown in Supplementary Table 1.

### Risk of bias assessment

The overall risk of bias within studies is acceptable, except for a few studies with a relatively high risk of bias (Supplementary Figure 2A). The overall risk of bias across studies is acceptable. For all the risk assessment indicators, more than 60% of those included literature were assessed as low risk (Supplementary Figure 2B).

### Synthesis of results

**Fig. 1:**
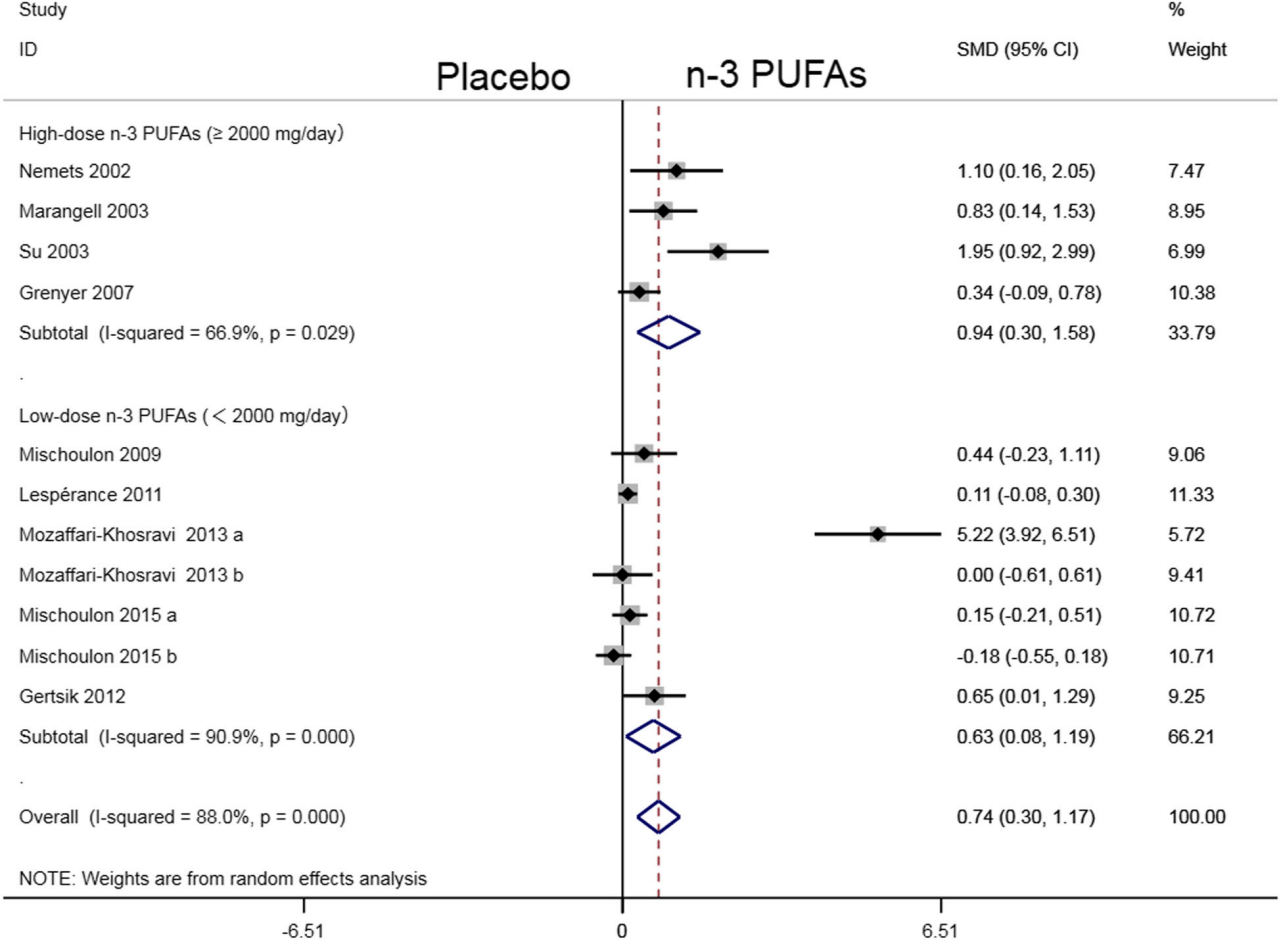
Forest plot

**Fig. 2:**
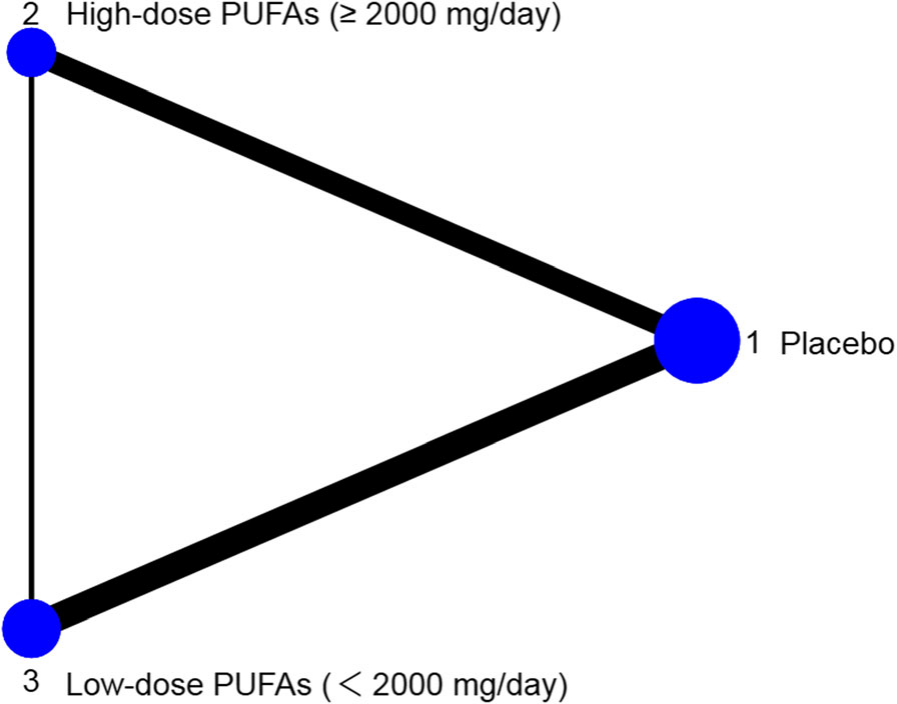
Network plot. Nodes represent the competing strategies which are weighted according to the total number of each study; edges represent the available direct comparisons between pairs of strategies which are weighted according to the standard error (se) of each study

**Fig. 3:**
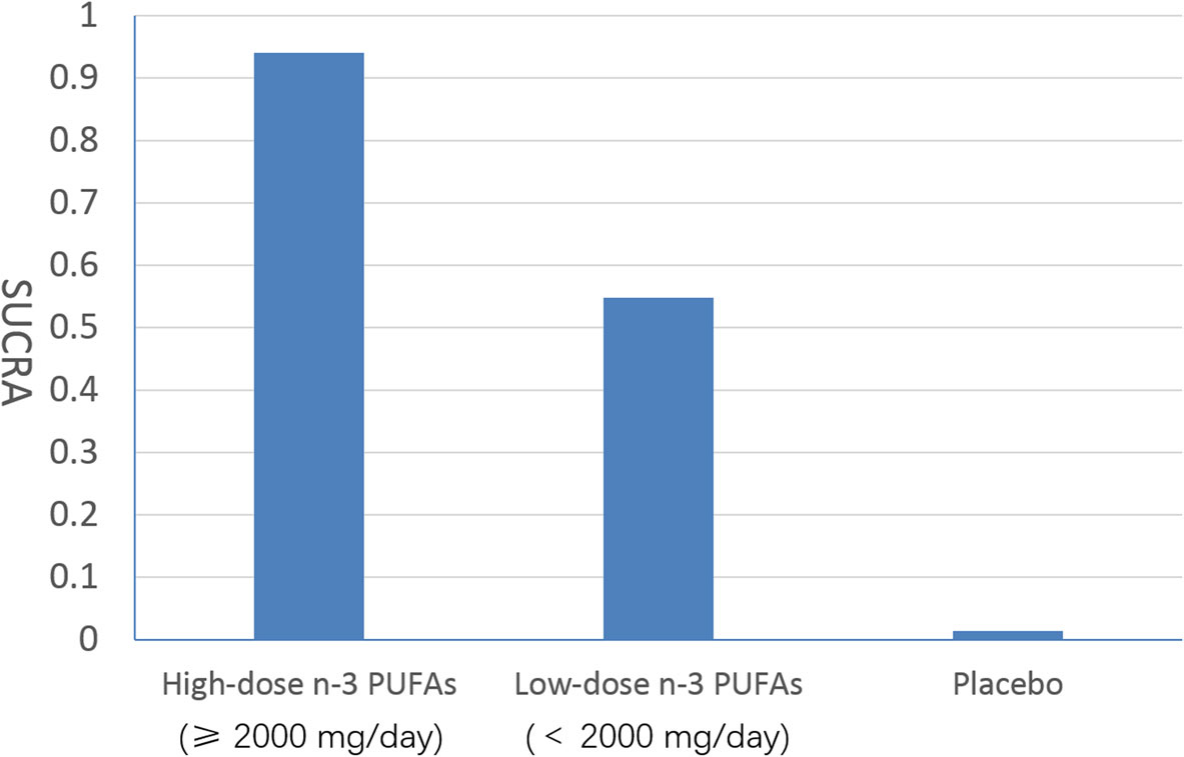
Rankogram

We synthesized the pooled evidence in a pairwise meta-analysis for 9 placebo-controlled trials and found that n-3 PUFAs were superior to placebo (SMD: 0.71; 95% CI: − 1.13 ~ − 0.28). Subgroup analysis showed that, both the high-dose n-3 PUFAs subgroup (SMD: -0.90; 95% CI: − 1.51 ~ − 0.29) and low-dose PUFAs subgroup (SMD: -0.61; 95% CI: − 1.15 ~ − 0.06) were superior to the placebo subgroup (Fig. 1). Results of the network meta-analysis for all the available data showed that, both high (SMD: 0.908 ± 0.331; 95% CI: 0.262 ~ 1.581) and low-dose (SMD: 0.601 ± 0.286; 95% CI: 0.034 ~ 1.18) n-3 PUFAs were superior to placebo (Table 1). The network plot was presented in Fig. 2. After pairwise comparisons in network meta-analysis, we ranked all the interventions by SUCRA: high-dose n-3 PUFAs: 0.939, low-dose n-3 PUFAs: 0.547, Placebo: 0.012. The best treatment strategy is the high-dose n-3 PUFAs strategy (Fig. 3).

**Table 1:** League table for network meta-analysis

	SMD*	Mean ± SD (95% CI)
1	[1, 2]	0.908 ± 0.331 (0.262, 1.581)
2	[1, 3]	0.601 ± 0.286 (0.034, 1.18)
3	[2, 3]	−0.307 ± 0.392 (− 1.097, 0.471)

* The numbers in brackets in the column marked with asterisk represent different treatment schemes: 1, Placebo; 2, High-dose n-3 PUFAs (≥ 2000 mg/day); 3, low-dose n-3 PUFAs (< 2000 mg/day)

## Additional analysis

### Heterogeneity analysis

The result of heterogeneity investigation showed that an *I*^*2*^ statistic of 87.4% (*P*<0.001) indicated a relatively high heterogeneity of all the included RCTs. In subgroup analysis, *I*^*2*^ statistics were 63.5% (*P* = 0.0042) and 90.4% (*P*<0.001) for the high- and low-dose n-3 PUFAs subgroup respectively (Fig. 1).

### Sensitivity analysis

Results of the sensitivity analysis indicated that the estimated values of our meta-analysis were basically within the confidence interval no matter which trial was withdraw from the pooled results, suggesting the results of the meta-analysis were stable (Supplementary Table 3, Supplementary Figure 3).

### Network meta-regression

Results of network meta-regression showed that the publication year, the dose of DHA administrated, time for administration, baseline severity and anti-depressants usage were not correlated with the estimated effect of the network meta-analysis (Supplementary Table 2, Supplementary Figure 4).

### Publication bias

The comparison-adjusted funnel plot for 9 placebo-controlled trials is shown in Supplementary Figure 5. Results of Egger’s test for small-study effects were − 3.649 ± 1.205 (95% CI: − 6.375 to − 0.922; *P* = 0.014), suggesting significant small-study effects existed. Therefore, we implemented trim-and-fill methods with random-effects model looking for missing studies and found that two studies needed to be supplemented, but results of meta-analysis before and after the missing studies supplementation showed little difference, which indicates that the results of our pairwise meta-analysis were robust (Supplementary Table 4.)

## Discussion

Results of our pairwise meta-analysis and network meta-analysis have shown a positive effect of n-3 PUFAs for MDD patients. We found that the efficacy of high-dose n-3 PUFAs is superior to that of low-dose. This result is consistent with our colleagues’ previous findings [7, 24]. They have found that treatments enriched with EPA increased levels of eicosapentaenoyl ethanolamide in plasma, which was positively associated with clinical remission [24]. As there are two active ingredients in the preparation, we had speculated the efficacy of n-3 PUFAs for MDD was affected by the quantity of DHA. Another finding of our colleagues [24] is that the EPA monotherapy and the EPA combined DHA therapy had significantly reduced the depressive symptoms compared to the DHA monotherapy, which is consistent with the finding of our network meta-regression and reinforced the notions that EPA might be the main antidepressant component. However, results of the network meta-regression showed the pooled evidence was uncorrelated to DHA dose. Whether the dose of DHA affects the efficacy of n-3 PUFAs needs further study.

EPA advantage doctrine has almost become a consensus. Compared with EPA, DHA has a longer carbon chain and additional double bonds, which have a greater impact on the fluidity, protein and ion channel activity of the cell membrane. Usually, n-3 PUFAs supplementations contain higher EPA than DHA. During the metabolic process, EPA experienced a more β-oxidized metabolic process than DHA do, which retaining in prototype [26] and limiting the efficacy. An exploratory hypothesis-testing meta-analysis performed in 35 RCTs including 11,038 participants with diagnosed depression found that, EPA-predominant formulations (> 50% EPA) demonstrated clinical benefits compared with placebo whereas DHA-predominant formulations (> 50% DHA) did not [11]. ISNPRP guidelines for n-3 PUFAs in the Treatment of MDD proposed that, both pure EPA or an EPA/DHA combination of a ratio higher than 2 (EPA/DHA > 2:1) are considered effective, and the recommended dosages should be 1000–2000 mg of net EPA daily, from either pure EPA or an EPA/DHA (> 2:1) formula. Our results of network meta-regression showed that the dose of DHA administrated was not correlated with the estimated effect of the network meta-analysis (Supplementary Table 2, Supplementary Figure 4). In other words, our results confirm the leading role of EPA in MDD treatment again. To this end, more clinical trials need to be performed to compare the efficacy of DHA with EPA. Because the content of EPA and DHA in n-3 PUFAs preparation is quite different.

In terms of the safety of high-dose n-3 PUFAs, we need to pay attention to its possible short-term and long-term adverse reactions. The first issue to be concerned about is the haemorrhage risk in short-term use. Some scholars believe that the risk of theoretical adverse effects of excessive bleeding did not exist, and current evidence suggests that under concurrent usage of antiplatelet or anticoagulant agents, doses up to 4 g of n–3 PUFAs daily are not associated with an increased risk of major bleeding [27]. An observation on healthy volunteers revealed that high-dose n-3 PUFAs (2520 mg) has no effect on platelet aggregation or coagulation measured with static and flow-based aggregation instruments and Sonoclot [28]. Recently, Guidelines also indicate that the theoretical risk of excessive bleeding from n-3 PUFAs does not exist, even if patients are using antiplatelet and anticoagulants [12]. People using n-3 PUFAs were more likely to have mild gastrointestinal symptoms (such as fishy smell, hiccups, and nausea), less skin abnormalities (such as rash, itching), and no serious side effects were reported [29]. A previous meta-analysis directly took side effects as one of the main observation indicators, and found that although the quality of the data was not good, the number of individuals with adverse reactions in the n-3 PUFAs group and the placebo group was equivalent, and both were negligible [5]. Recently, some trials applied higher dosages of n-3 PUFAs (3–6 g/day) and no serious adverse effect were found [30–35]. Additionally, by considering that pregnant women can be administrated in a high dose [36], we tentatively infer the safety of n-3 PUFAs is acceptable in the short term. The second issue to be concerned about is the long-term adverse effect. There had been found that more than 1000 mg/day of EPA will result in more adverse effects and less of augmentation effect for antidepressants [37]. Previous studies reveal that, the effective antidepressive dosage among Taiwanese ranges from 2.2 to 4.4 g/day for EPA and from 1.2 to 2.2 g/day for DHA [7, 36, 38]. But these observations did not last more than 8 weeks, which lack of evidence for long-term observation. Available evidence suggests that 4 g/day of n-3 PUFAs were safe and well tolerated for up to 52 weeks of treatment in Japanese patients with hypertriglyceridemia undergoing lifestyle modification [39]. So we initially speculate that it is safe for long-term use in patients with MDD, but studies with long-term follow up need to be performed to acquired more reliable evidence for high-dose n-3 PUFAs in MDD adjuvant therapeutic, as a previous systematic review proposed that the post-marketing surveillance and observational studies are still necessary to identify long-term adverse effects and to confirm the safety and tolerability profiles of the prescription omega-3 fatty acid products [29].

The determination of the cut-off point for high and low dose subgroups has not yet been determined. Prior to determining high doses of n-3 PUFAs ≥2000 mg, we have tried different grouping methods (data not shown). These grouping methods include high-medium-low-dose subgroups and EPA monotherapy subgroups. Evidence of heterogeneity or merger is not ideal. Therefore, we reviewed some clinical trials on n-3 PUFAs for depression and EPA pharmacokinetic studies in healthy subjects. In clinical applications, fish oil preparations have different definitions of high doses due to different purity and different regions. A recent research defined 3 g/day of high-purity (90%) n-3 PUFAs preparation as high-dose, which is equivalent to that of ordinary preparation of 1.8 g/d in Japan due to the very high dietary intake of n-3 PUFAs [40]. Studies on healthy subjects have shown that a daily intake of more than 2 g of high-purity EPA can increase EPA concentrations in plasma and red blood cells in a dose-dependent manner [41]. The latest ISNPRP guidelines use pure EPA 1–2 g as the recommended dose [12]. Based on the above factors, we finally determined a dose of ≥2000 mg/day as a high dose.

It is controversial that the effect of n-3 PUFAs on depression remains to be determined by further research, due to the heterogeneity of clinical studies. A previous meta-analysis [42] also demonstrated significant heterogeneity and publication bias. Nearly all evidence of n-3 PUFAs benefit was removed after adjusting for publication bias by using the trim-and-fill method. That study also demonstrated no significant difference in the efficacy of n-3 PUFA in trials based on the dose of DHA and EPA utilized. For further heterogeneity reducing, we have excluded the MDD participants with co-morbid conditions. Our results showed that heterogeneity was obviously in both high- and low-dose n-3 PUFAs subgroups. Sensitivity analysis revealed that the included literature had no effect on the overall results of the pairwise meta-analysis. According to the results of published bias assessment, although there are small sample studies and missing studies, there is no significant change in the overall results of pairwise meta-analysis after supplementing the missing studies with the trim-and-fill method. In addition, the results of network meta-regression showed that there was no correlation between the covariates and the efficacy of each subgroup. Therefore, we have reason to suspect that heterogeneity may originate from the different proportions or purity of components in n-3 PUFAs formulation. Some scholars argued that EPA has synergistic effects with DHA in combined treatments of depression [43].

The difference between this study and previous meta-analysis is that we conducted direct and indirect comparisons of n-3 PUFAs at different dose levels while minimizing heterogeneity (such as including MDD patients without comorbidities, subgroup analysis considering heterogeneity, etc.), while applying network-regression analysis to examine multiple Effects of covariates on high and low dose efficacy. Combined with the results of other previous studies [41, 44], it has been clarified that there is a dose-effect mechanism for n-3 PUFAs in the treatment of MDD. The universality of our results is limited to eligible individuals who are included in clinical trials such as MDD patients without any comorbidity. Our conclusion is unnecessarily related to most patients with mild-to-moderate depression with or without comorbidities. In addition, n-3 PUFAs supplementation for MDD may vary depending on age, gender, and previous antidepressant treatment, and these cannot be explored by using the data from clinical trials included in this study.

## Limitations

This paper only focuses on the efficacy of the n-3 PUFAs, but do not pay attention to the adverse effects, especially the long-term adverse effects after high-dose treatment, which need to be further studied in the future. The limitation of this study was also attributed to the small number of eligible RCTs, which limit the power of the network meta-analysis. Meanwhile, the heterogeneity of the included literature is obvious. Despite efforts to explore, we still can not find its source and improve it, so the interpretation of our results should be rigorous.

## Conclusions

High-dose n-3 PUFAs supplementation might be more superior than low-dose in the early therapy period for MDD. More head-to-head clinical trials need to be carried out to provide more direct comparison and enhance the evidence of the efficacy of n-3 PUFAs for MDD. At the same time, the comparison between EPA and DHA needs to be strengthened to further clarify the difference between dosage and efficacy between the two components of n-3 PUFAs.

## Supplementary information

**Additional file 1: Figure S1.** flow diagram. **Figure S2.** Risk of bias. **Figure S3.** sensitivity analysis. **Figure S4.** network meta-regression. **Figure S5.**-comparison adjusted funnel plot. **Table S1.** study characteristic. **Table S2.** Results of network meta-regression. **Table S3.** Sensitivity analysis. **Table S4.** Results of publication bias assessment with trim and fill method.

## Data Availability

All data generated or analyzed during this study are included in this published article and the supplementary material.

## Abbreviations

n-3 PUFAs: n-3 Polyunsaturated Fatty Acids
MDD: Major Depressive Disorder
DHA: Docosahexaenoic Acid
EPA: Eicosapentaenoic Acid
ISNPRP: International Society for Nutritional Psychiatry Research Practice
RCTs: Randomized Controlled Trials
SMD: Standardized Mean Deviations
CI: Confidence Interval
SUCRA: Surface Under The Cumulative Ranking Curve
