# Supplementary material for: High-dose omega-3 polyunsaturated fatty acid supplementation might be more superior than low-dose for major depressive disorder in early therapy period: a network meta-analysis
Source: BMC Psychiatry. 2020 May 20;20:248. doi: 10.1186/s12888-020-02656-3 (PMC7238659; doi:10.1186/s12888-020-02656-3)
Supplement: Supplementary file 1 — Additional file 1: Figure S1. flow diagram. Figure S2. Risk of bias. Figure S3. sensitivity analysis. Figure S4. network meta-regression. Figure S5.-comparison adjusted funnel plot. Table S1. study characteristic. Table S2. Results of network meta-regression. Table S3. Sensitivity analysis. Table S4. Results of publication bias assessment with trim and fill method. [file 12888_2020_2656_MOESM1_ESM.docx]

High-dose omega-3 polyunsaturated fatty acid supplementation might be more superior than low-dose for major depressive disorder in the early therapy period: a network meta-analysis

Supplementary materials

Supplementary Figure 1. flow diagram


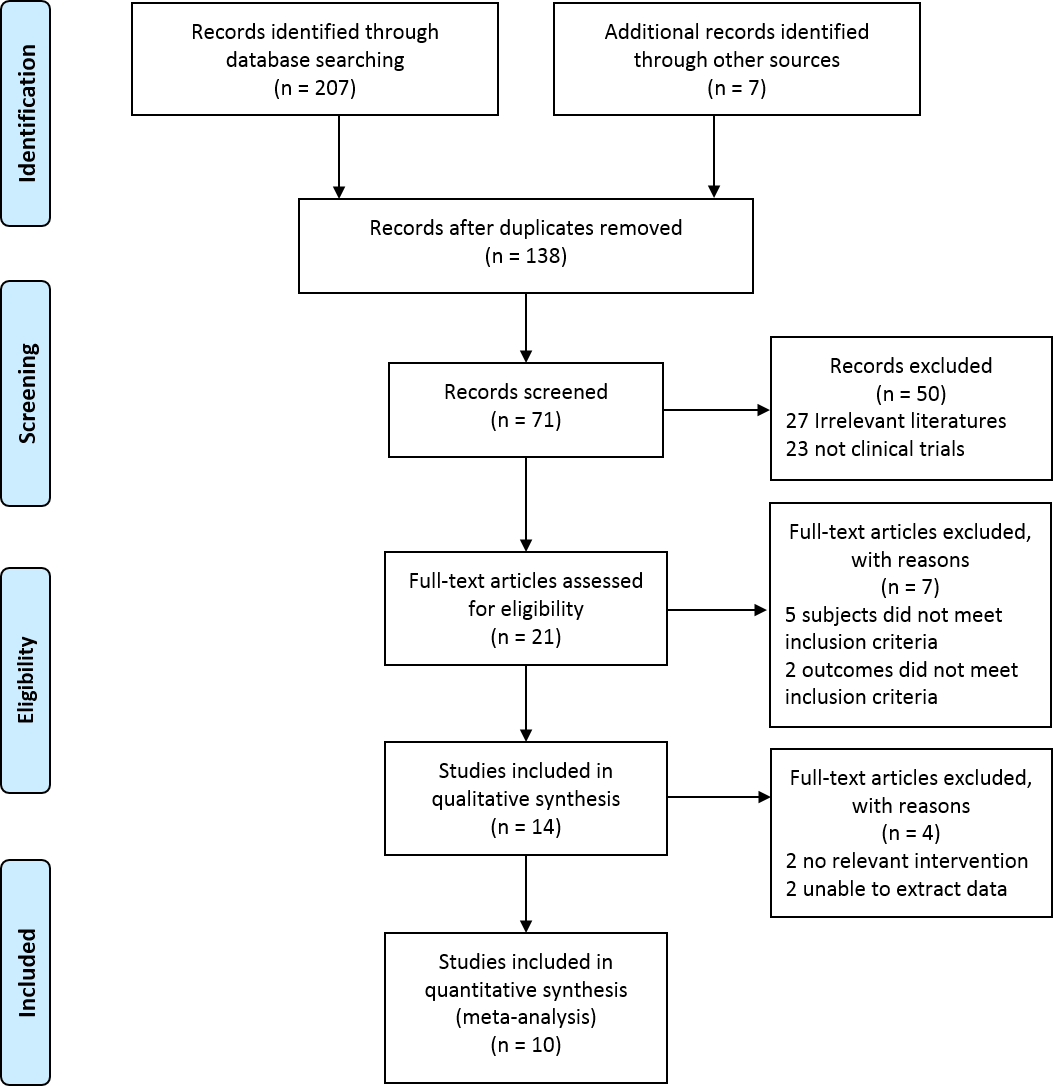


Supplementary Figure 2. Risk of bias

A


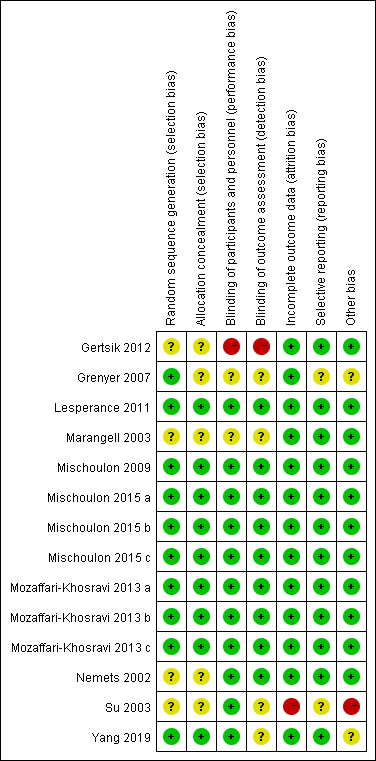


Risk of bias summary: each risk of bias item for each included study.

B


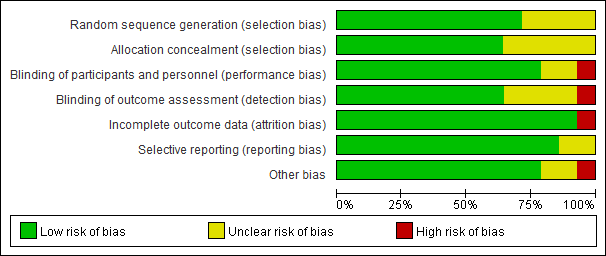


Risk of bias graph: each risk of bias item presented as percentages across all included studies.

Supplementary Figure 3 sensitivity analysis

Supplementary Figure 3.1 sensitivity analysis for high-dose PUFAs (≥ 2000 mg/day) subgroup


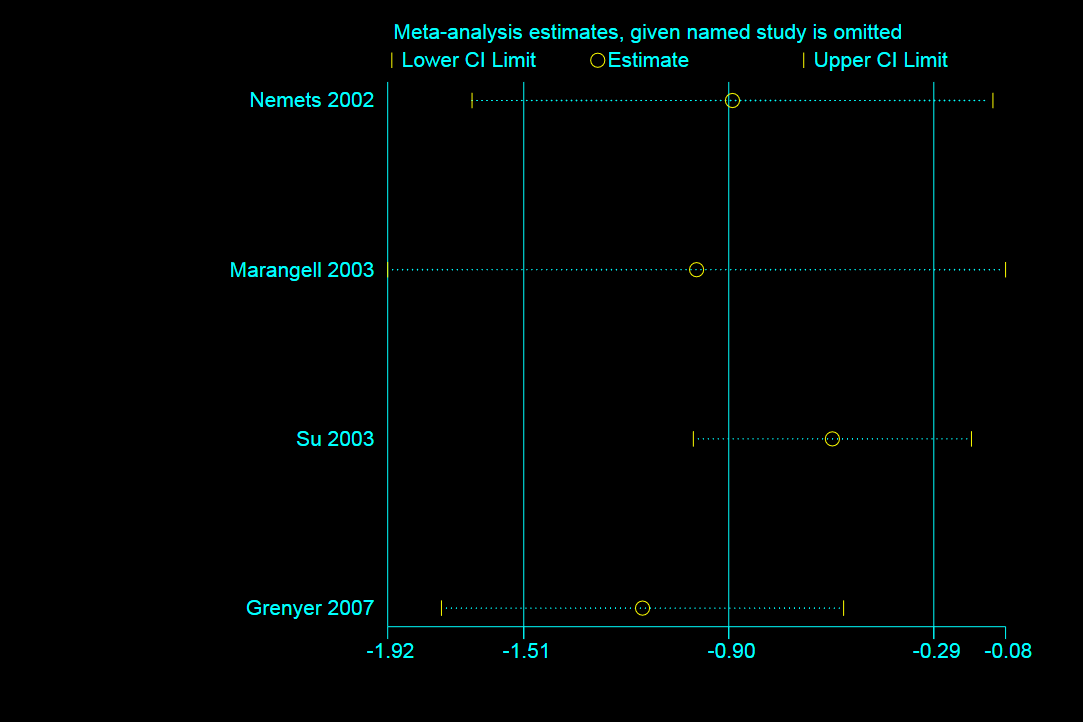


Supplementary Figure 3.1 sensitivity analysis for low-dose PUFAs (＜ 2000 mg/day) subgroup


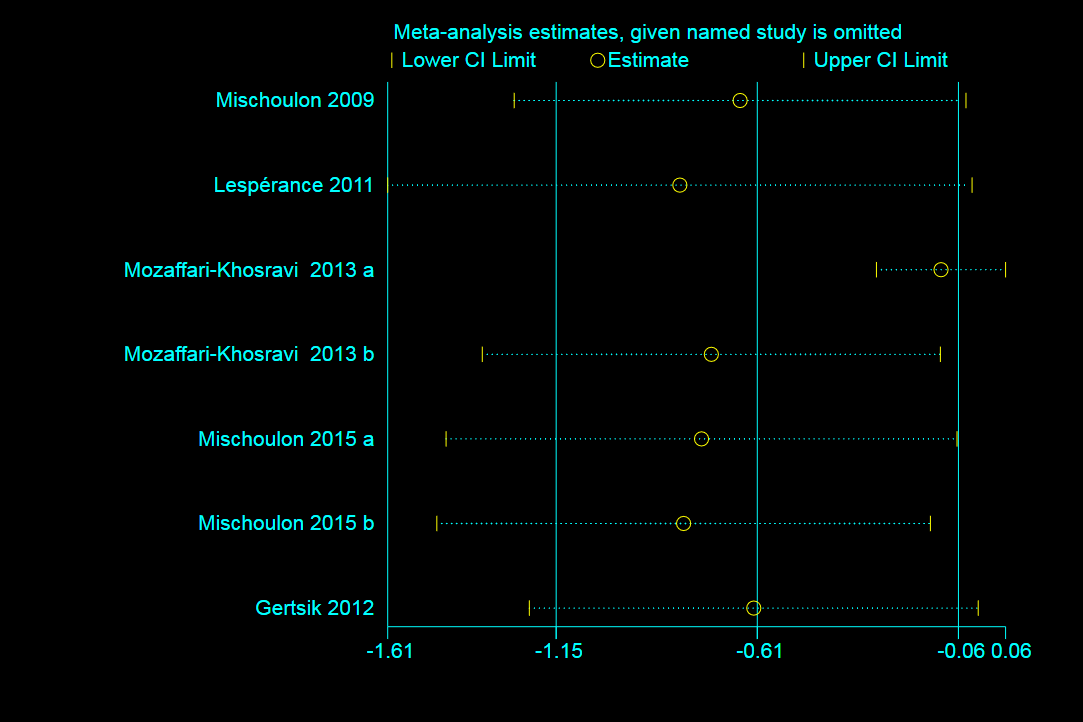


Supplementary Figure 4 network meta-regression

(PUFAs_hi: ≥ 2000 mg/day; PUFAs_lo: ＜ 2000 mg/day）

Supplementary Figure 4.1 treatment effect vs publication year in different subgroup


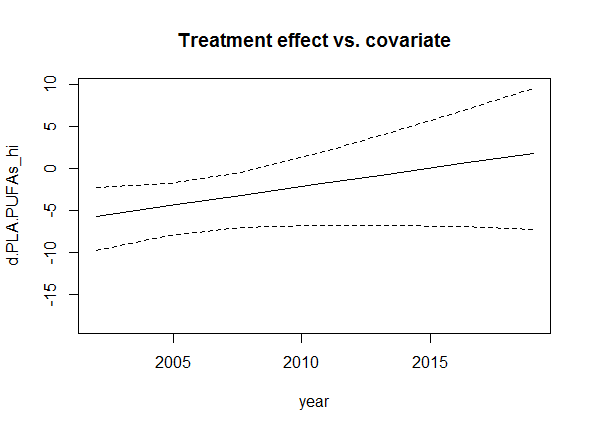

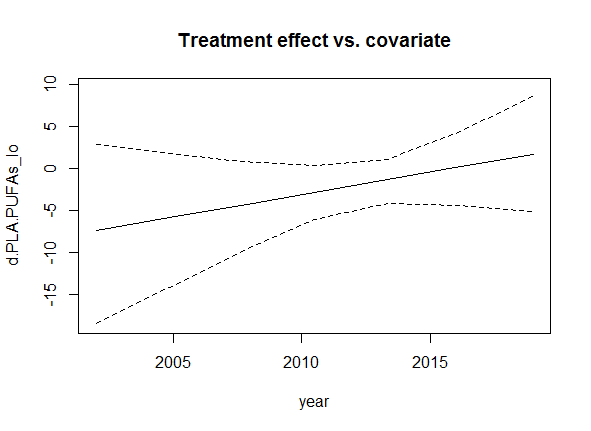


Supplementary Figure 4.2 treatment effect vs treatment time in different subgroup


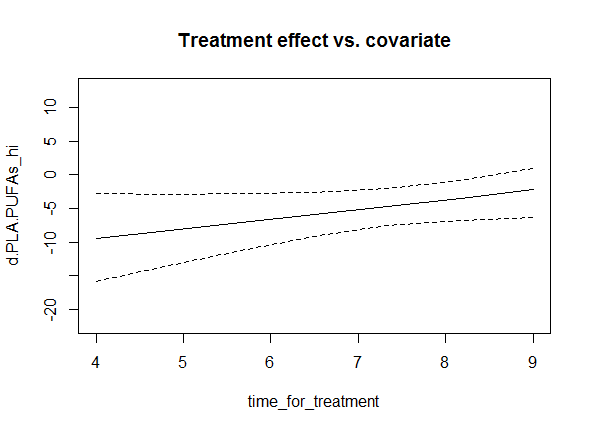

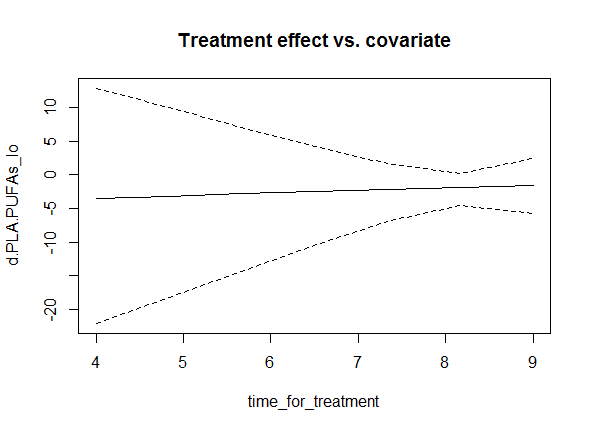


Supplementary Figure 4.3 treatment effect vs DHA dose in different subgroup


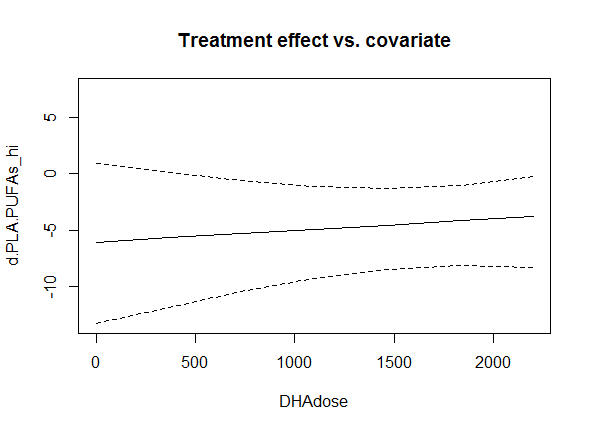

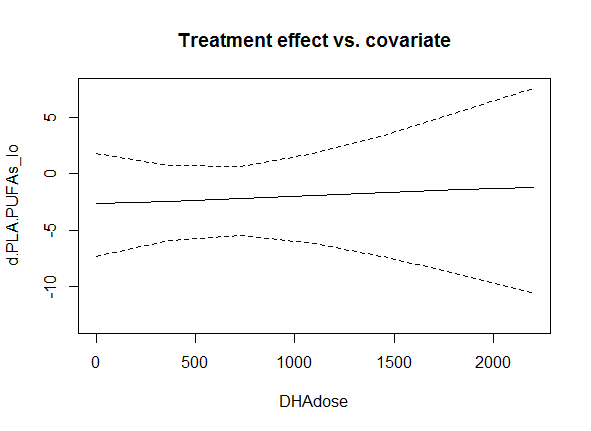


Supplementary Figure 4.4 treatment effect vs antidepressant usage in different subgroup


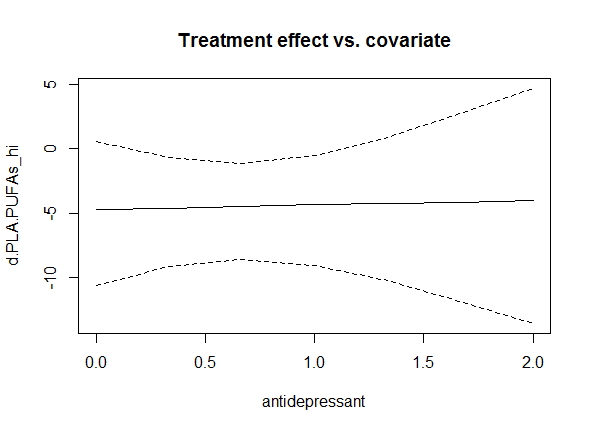

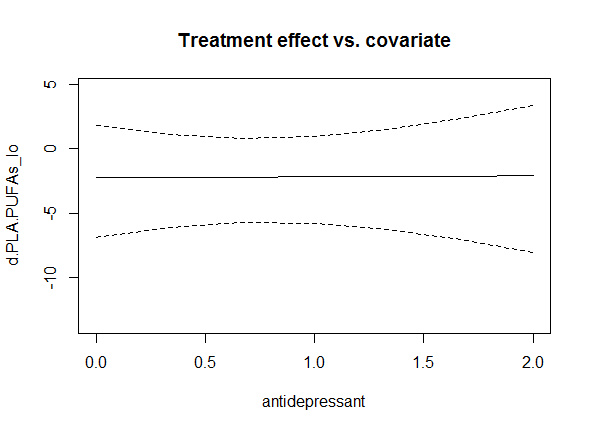


Supplementary Figure 4.5 treatment effect vs baseline severity in different subgroup


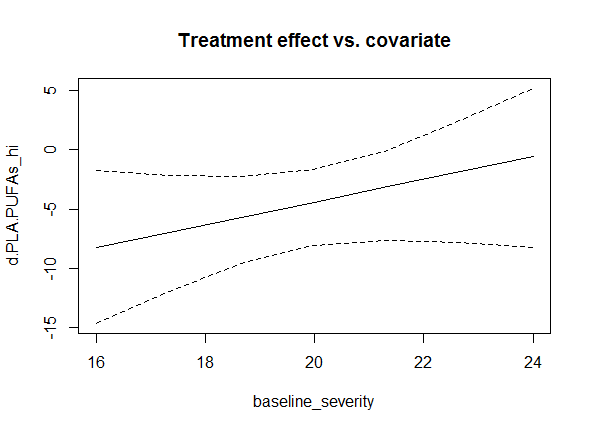

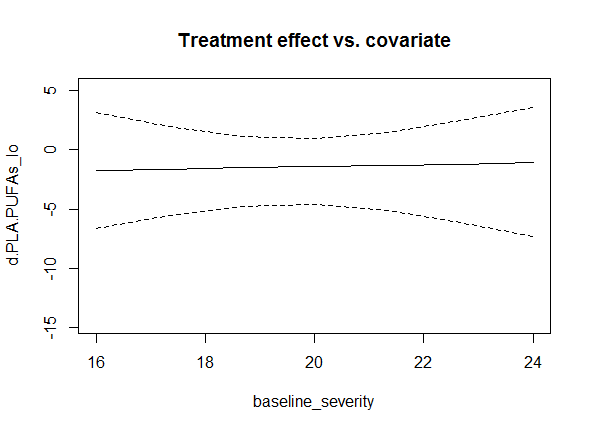


Supplementary Figure 5-comparison adjusted funnel plot


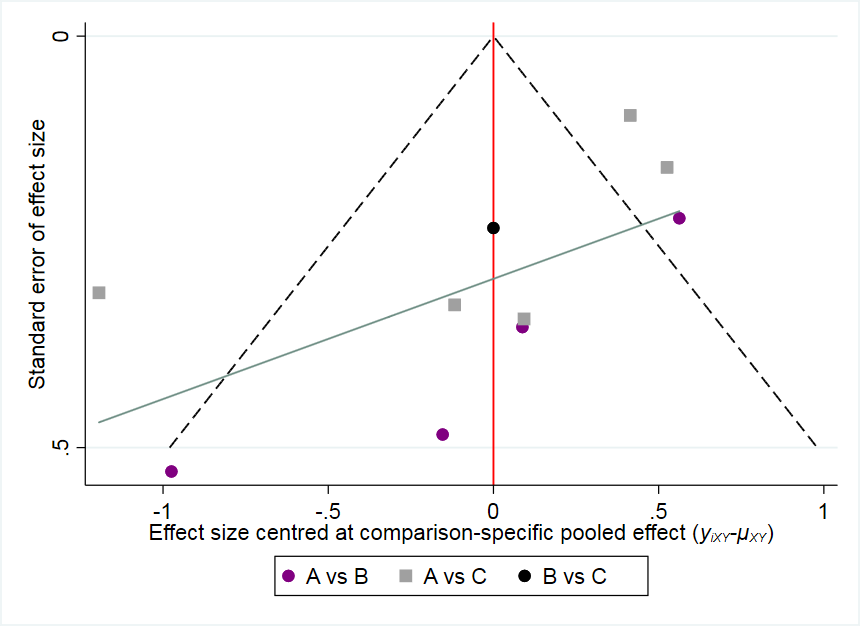


A: Placebo, B: High-dose n-3 PUFAs (≥ 2000 mg/day), C: low-dose n-3 PUFAs (＜ 2000 mg/day).

Supplementary Table 1 study characteristic

| Study name | Year | Comparison | Dose EPA (mg/day) | Dose DHA (mg/day) | Outcome | Baseline severity | Total N | Treatment time(wk) |
| --- | --- | --- | --- | --- | --- | --- | --- | --- |
| Nemets | 2002 | EPA vs PLA | 2000 | 0 | HDRS-24 | 17 | 20 | 4 |
| Marangell | 2003 | DHA vs PLA | 0 | 2000 | MADRS | 19 | 35 | 6 |
| Su | 2003 | DHA+EPA vs PLA | 4400 | 2200 | HDRS-21 | 19 | 22 | 8 |
| Grenyer | 2007 | DHA+EPA vs PLA | 600 | 2200 | HDRS-21 | 21 | 83 | 9 |
| Mischoulon | 2009 | EPA vs PLA | 1000 | 0 | HDRS-17 | 20 | 35 | 8 |
| Lespérance | 2011 | DHA+EPA vs PLA | 1050 | 150 | IDS-SR30 | 21 | 432 | 9 |
| Mozaffari-Khosravi | 2012 | DHA vs PLA | 1000 | 1000 | HDRS-17 | 16 | 41 | 8 |
| Mischoulon | 2015 | DHA vs EPA vs PLA | 1000 | 1000 | HDRS-17 | 20 | 117 | 8 |
| Gertsik | 2012 | DHA+EPA vs EPA vs PLA | 1800 | 400 | HDRS-21 | 22 | 40 | 8 |
| Yang | 2019 | DHA vs EPA vs PLA | 3000 | 1400 | HDRS-21 | 24 | 85 | 8 |

Supplementary Table 2 Results of network meta-regression

|  | Covariant | effect | Regression Coefficient | |
| --- | --- | --- | --- | --- |
|  |  |  | M ± SD | 95% CI |
| 1 | Antidreppressant | PLA vs PUFAs_hi | 0.510 ± 4.223 | -7.943 ~ 8.939 |
|  |  | PLA vs PUFAs_lo | 0.238 ± 2.536 | -4.637 ~ 5.518 |
| 2 | DHA dose | PLA vs PUFAs_hi | 1.671 ± 3.452 | -5.085 ~ 8.653 |
|  |  | PLA vs PUFAs_lo | 1.236 ± 4.746 | -8.383 ~ 10.825 |
| 3 | Publication year | PLA vs PUFAs_hi | 4.592 ± 3.262 | -2.089 ~ 10.878 |
|  |  | PLA vs PUFAs_lo | 5.201 ± 5.549 | -5.899 ~ 16.093 |
| 4 | baseline severity | PLA vs PUFAs_hi | 4.331 ± 3.412 | -2.713 ~ 10.745 |
|  |  | PLA vs PUFAs_lo | 0.278 ± 2.530 | -5.347 ~ 4.913 |
| 5 | Time for treatment | PLA vs PUFAs_hi | 4.182 ± 2.570 | -1.0801 ~ 9.1027 |
|  |  | PLA vs PUFAs_lo | 1.162 ± 6.143 | -10.581 ~ 14.040 |

(PUFAs_hi: ≥ 2000 mg/day; PUFAs_lo: < 2000 mg/day)

Supplementary Table 3. Sensitivity analysis

Supplementary Table 3.1

Sensitivity analysis for high-dose PUFAs (≥ 2000 mg/day) subgroup

| Study omitted | Estimate | [95% Conf. Interval] |
| --- | --- | --- |
| Nemets 2002 | -0.92884582 | -1.7391479 ~ -0.1185438 |
| Marangell 2003 | -1.0448818 | -2.0085251 ~ -0.08123838 |
| Su 2003 | -0.62468022 | -1.0655291 ~ -0.18383141 |
| Grenyer 2007 | -1.2108881 | -1.8418628 ~ -0.57991338 |
| Combined | -0.94428933 | -1.583626 ~ -0.30495264 |

Supplementary Table 3.2

Sensitivity analysis for high-dose PUFAs (< 2000 mg/day) subgroup

| Study omitted | Estimate | [95% Conf. Interval] |
| --- | --- | --- |
| Mischoulon 2009 | -0.70416731 | -1.4142603 ~ 0.0059257 |
| Lespérance 2011 | -0.90995324 | -1.8638531 ~ 0.04394668 |
| Mozaffari-Khosravi 2013 a | -0.07924119 | -.22321329 ~ 0.06473092 |
| Mozaffari-Khosravi 2013 b | -0.80105948 | -1.5235479 ~ -0.07857099 |
| Mischoulon 2015 a | -0.83854407 | -1.6602865 ~ -0.01680158 |
| Mischoulon 2015 b | -0.89862776 | -1.6949005 ~ -0.102355 |
| Combined | -0.6418055 | -1.2620306 ~ -0.02158037 |

Supplementary Table 4 Results of publication bias assessment with trim and fill method

Meta-analysis

| Pooled 95% CI Asymptotic No. of

Method | Est Lower Upper z_value p_value studies

-------+----------------------------------------------------

Fixed | -0.244 -0.372 -0.116 -3.725 0.000 11

Random | -0.709 -1.133 -0.284 -3.273 0.001

Test for heterogeneity: Q= 79.057 on 10 degrees of freedom (p= 0.000)

Moment-based estimate of between studies variance = 0.400

Trimming estimator: Linear

Meta-analysis type: Random-effects model

iteration | estimate Tn # to trim diff

----------+--------------------------------------

1 | -0.709 39 1 66

2 | -0.836 42 2 6

3 | -0.949 46 2 8

4 | -0.949 46 2 0

Filled

Meta-analysis

| Pooled 95% CI Asymptotic No. of

Method | Est Lower Upper z_value p_value studies

-------+----------------------------------------------------

Fixed | -0.502 -0.621 -0.383 -8.290 0.000 13

Random | -0.989 -1.511 -0.468 -3.717 0.000

Test for heterogeneity: Q= 186.949 on 12 degrees of freedom (p= 0.000)

Moment-based estimate of between studies variance = 0.805

Supplementary Method 1. Literature search strategy

(1) Pubmed

((((((((((Depression) OR Depressions) OR Depressive Symptom) OR Depressive Symptom) OR Symptom, Depressive) OR Symptom, Depressive) OR Emotional Depression) OR Depression, Emotional) OR Depression, Emotional) OR Emotional Depression) AND (((((((((((((((((((Fatty Acids, Omega 3) OR n3 Fatty Acid) OR Fatty Acid, n3) OR n3 PUFA) OR PUFA, n3) OR n3 Polyunsaturated Fatty Acid) OR n3 Oils) OR Oils, n3) OR n-3 PUFA) OR Omega-3 Fatty Acids) OR n-3 Fatty Acids) OR Fatty Acids, n-3) OR n 3 Fatty Acids) OR n-3 Polyunsaturated Fatty Acid) OR n 3 Polyunsaturated Fatty Acid) OR Omega 3 Fatty Acids) OR n-3 Oils) OR Oils, n-3) OR n 3 Oils) AND (((((preventive therapy) OR prophylaxis) OR preventive measures) OR prevention) OR control) AND ((((RCT[Title/Abstract]) OR RCTS[Title/Abstract]) OR random[Title/Abstract]) OR blind[Title/Abstract]) Filters: Clinical Trial

(2) Embase

#1. 'fatty acids, omega 3'/exp OR 'fatty acids, omega 3' OR 'n3 fatty acid' OR 'fatty acid, n3' OR 'n3 pufa' OR 'pufa, n3' OR 'n3 polyunsaturated fatty acid' OR 'n3 oils' OR 'oils, n3' OR 'n-3 pufa' OR 'omega-3 fatty acids' OR 'n-3 fatty acids' OR 'fatty acids, n-3' OR 'n 3 fatty acids' OR 'n-3 polyunsaturated fatty acid'/exp OR 'n-3 polyunsaturated fatty acid' OR 'n 3 polyunsaturated fatty acid'/exp OR 'n 3 polyunsaturated fatty acid' OR 'omega 3 fatty acids' OR 'n-3 oils' OR 'oils, n-3' OR 'n 3 oils'

#2. 'depression' OR 'depressions' OR 'depressive symptoms' OR 'depressive symptom' OR 'symptom, depressive' OR 'emotional depression' OR 'depression, emotional' OR 'depressions, emotional' OR 'emotional depressions'

#3. 'preventive therapy' OR 'prophylaxis' OR 'preventive measures' OR 'prevention' OR 'control'

#4. [controlled clinical trial]/lim OR [randomized controlled trial]/lim

#5 #1 AND #2 AND #3 AND #4

(3) the Cochrane Library

#1 (Depression):ti,ab,kw OR (Depressions):ti,ab,kw OR (Depressive Symptoms):ti,ab,kw OR (Depressive Symptom):ti,ab,kw OR (Symptom, Depressive):ti,ab,kw OR (Symptoms, Depressive):ti,ab,kw OR (Emotional Depression):ti,ab,kw OR (Depression, Emotional):ti,ab,kw OR (Depressions, Emotional):ti,ab,kw OR (Emotional Depressions):ti,ab,kw

#2 (Fatty Acids, Omega 3):ti,ab,kw OR (n3 Fatty Acid):ti,ab,kw OR (Fatty Acid, n3):ti,ab,kw OR (n3 PUFA):ti,ab,kw OR (PUFA, n3):ti,ab,kw OR (n3 Polyunsaturated Fatty Acid):ti,ab,kw OR (n3 Oils):ti,ab,kw OR (Oils, n3):ti,ab,kw OR (n-3 PUFA):ti,ab,kw OR (Omega-3 Fatty Acids):ti,ab,kw OR (n-3 Fatty Acids):ti,ab,kw OR (Fatty Acids, n-3):ti,ab,kw OR (n 3 Fatty Acids):ti,ab,kw OR (n-3 Polyunsaturated Fatty Acid):ti,ab,kw OR (n 3 Polyunsaturated Fatty Acid):ti,ab,kw OR (Omega 3 Fatty Acids):ti,ab,kw OR (n-3 Oils):ti,ab,kw OR (Oils, n-3):ti,ab,kw OR (n 3 Oils):ti,ab,kw

#3 (preventive therapy):ti,ab,kw OR (prophylaxis):ti,ab,kw OR (preventive measures):ti,ab,kw OR (prevention):ti,ab,kw

#4 #1 and #2 and #3
